# Mediating effects of depressive symptoms on social support and quality of life among rural older Chinese

**DOI:** 10.1186/s12955-020-01490-1

**Published:** 2020-07-20

**Authors:** Jiayu Wang, Jiang Xue, Yuxing Jiang, Tingfei Zhu, Shulin Chen

**Affiliations:** Department of psychology and behavioral sciences, Zhejiang University, 148 Tianmushan Road, Hangzhou, 310028 China

**Keywords:** Late-life depression, Social support, Quality of life, Mediation

## Abstract

**Background:**

It is not well clear how psychosocial factors like depressive symptoms, social support affect quality of life in rural elderly in China. This study aimed to investigate the mediating role of depressive symptoms in the association between social support and quality of life.

**Methods:**

Cross-sectional data of 420 rural elderly were taken from four villages in Hangzhou City. They were interviewed with a demographic questionnaire, the Patient Health Questionnaire (PHQ-9) for depression, the Medical Outcomes Study Social Support Survey (MOS-SSS) for social support, and the short version of World Health Organization Quality of Life Assessment (WHOQOL-BREF) for quality of life. Mediation was examined by a nonparametric Bootstrapping method, controlling for socioeconomic variables.

**Results:**

Poor quality of life was associated with low social support and increased depressive symptoms. A significant indirect effect of social support existed through depression in relation to quality of life (ab = 0.0213, 95% CI [0.0071, 0.0421]), accounting for 9.5% of the effect of social support on quality of life. Approximately 4.8% of the variance in QOL was attributable to the indirect effect of social support through depressive symptoms.

**Conclusions:**

Depressive symptoms mediated the impact of social support on quality of life among rural older adults.

## Introduction

There were approximately 150 million Chinese residents aged 65 years and above in 2017, accounting for 11.4% of China’s total population informed by National Bureau of Statistics in 2018 (http://www.stats.gov.cn/tjsj/ndsj/2018/indexeh.htm), and more than 70% of the aging population is distributed in rural areas [1]. As the population ages, the number of rural elderly populations will continue to increase due to the increasing life expectancy. The demographic transition presents challenges to health authorities, especially in terms of the increasing burden of disease and its negative effect on quality of life (QOL) of older adults. Increasing life expectancy without improving QOL directly influences health expenditures and has become a key public health issue in the more developed countries [2, 3]. It may also become a major burden to developing countries with high population densities and emerging economies, such as China and India. Actually in China, improving the QOL and physical and mental health of the elderly in rural areas is the basic guarantee for achieving the goal of healthy aging and active aging in China.

QOL is a multidimensional concept related to an individual’s satisfaction with various aspects of life, such as physical, psychological, social, environmental and general health perceptions. As indicated in some studies, QOL of older people in rural China is low [4, 5]. Older adults in countryside had poorer QOL and lower subjective well-being than the those live in town [6]. More than 16% of rural elderly people were still in poor health condition and about 12% often felt lonely and majority of them were lacking of entertainment activities [1]. QOL of elderly people is still poor and worry, especially continuing massive rural-to-urban migrations of mostly young adults and leaving more rural elderly in the village.

Social support has been recognized as a crucial role in improving QOL. It was identified four dimensions by Sherbourne and Stewart [7]: (1) emotional/ informational support (expression of positive affect, empathetic understanding, and encouragement of expressions of feelings/ offering of advice, information, guidance or feedback); (2) tangible support (provision of material aid or behavioral assistance); (3) positive social interaction (availability of other persons to do fun things with you); (4) affectionate support (involving expressions of love and affection). In the stress-buffering model, social support is supposed to increase an individual’s positive emotions to cushion the negative effects of stress [8, 9]. A number of studies have shown that social support has a positive impact on the QOL of elderly people [10–12]. Additionally, older people with sufficient social support tend to report higher life satisfaction score [13, 14] and psychological well-being [15]. There is no doubt that social support is benefit to QOL. However, the pathway how social support influences QOL in aging population is still unknown.

For aging people, depression is one of the most common psychiatric disorders [16]. Several studies have indicated that depressive symptoms were important predictors of QOL in the elderly [12, 17–19]. QOL scores were low in the presence of depressive symptoms among community-dwelling older adults [20] and the presence of minor symptoms of depression contributed the greatest amount of variance to the vast majority of QOL measures [21].

Depressed older people are more likely to have low social support as proven in earlier studies [12, 22–24]. A meta-analysis illustrated that older adults with more social support had lower prevalence of depression [25]. Lack of social support and feelings of loneliness are believed to be risk factors for depression in the elderly [26, 27] and perceived social support has been negatively associated with late-life depressive symptoms [28]. Also, living alone and decreased social participation and engagement reduce positive emotions of an older individual [29–31].

It seems plausible that depressive symptoms of older people are likely to account for poor QOL of older people with low social support. As Bekele et al. [32] proposed, a perceived lack of social support increases perceived threats of stressful events, and this leads to an increase in depressive symptoms and influences QOL ultimately. Social support is an important resource and the limited support of family and friends is one of the issues that affect the lives of older adults. Those who are living alone or empty-nest older adults are likely to be more vulnerable to depression [30, 33], and then have a worse QOL. The possible mediation effect of depressive symptoms on the association between social support and QOL enlightens us to give more attention to older adults with low social support.

Although the interactions between social support, depressive symptoms, and QOL have strong evidences, few of studies provide direct evidence for depressive symptoms as a pathway linking social support with QOL in rural Chinese older people. Thence, this study aims to clarify the direct and indirect effects of social support and depressive symptoms on QOL in Chinese rural elderly. We investigated the hypothesis that depressive symptoms mediates the influence of social support on QOL in rural elderly.

## Methods

### Setting and participants

As the depressive symptoms scores were designed to be measured as a mediator, the sample size was calculated according to the liner multiple regression. We performed a priori of power analysis by G*Power. Assuming α of 0.05, power of 0.95 (generally required largest sample size), effect size of 0.15 and with 2 tested predictors, the output parameters showed that the total sample size was 107. Because our research was also an assessment service for the local elderly and we expected as many older adults as possible can be evaluated, four villages were randomly selected as sample sites.

A cross-sectional study was conducted in a rural county of Hangzhou. We used a multistage sampling method. All of the 16 towns in the rural county were numbered in list, and then random sampling were conducted with the number of samples setting as two by the Excel’s sampling analysis. Then, 2 villages in each town were randomly selected in the same way. Participants were eligible to participate if they were (1) community-dwelling residents registered to the selected villages; (2) aged 60 years old and above; and (3) capacity to communicate independently with interviewers. In each village, 120 elderly adults registered in their electronic health records were randomly selected by the Excel’s sampling analysis to be invited to participate in this study. Written informed consent was provided by 429 participants, of whom nine had missing data on relevant measurements. The remaining 420 were included for analyses.

### Measures

#### Depression

Depression was measured by the Chinese version of Patient Health Questionnaire (PHQ-9), which had been validated for use in the rural communities [34]. It is not just a screening tool for depression but also used to monitor the severity of depression [35]. The Cronbach’s α in this study was 0.653.

#### Social support

Social support was assessed by the Chinese version of Medical Outcomes Study Social Support Survey (MOS-SSS (C)) [36], which was shown to display good reliability and validity for non-clinical samples [37]. The Cronbach’s α was 0.902 for the total scale and the values for Cronbach’s α were 0.719–0.776 for dimensions in this study.

#### Quality of life

QOL was assessed using the Chinese version of World Health Organization Quality of Life Assessment (WHOQOL-BREF) [38, 39]. The instrument is composed of one item for general QOL (G1), one item for general health (G2) and 24 items from physical health, psychological health, social relationships, and environment domains. Domain scores were calculated by multiplying the mean of all item scores of each domain by a factor of 4 respectively, and potential scores for each domain ranged from 4 to 20 [40]. The overall QOL score is a total of four domains scores. The Cronbach’s α in this study was 0.839.

#### Demographic variables

A brief self-report measure was used to collect background information, including age, gender, education, marital status, and economic satisfactory.

Because of the low education attainment in the aging population, the questionnaires including PHQ-9, MOS-SSS, and WHOQOL-BREF were dictated by well-trained study investigators to the participants. After finishing all the questionnaires, each participant received a gift as a token of appreciation. It took about 30–45 min per participant for the whole assessment. The research was approved by the institutional review boards of Department of Psychology and Behavioral Sciences, Zhejiang University and supported by the project “The Construction of the Psychological Environment for Home Care” of Department of Civil Affairs of Zhejiang Province (No. ZMZD201507).

### Data analysis

Descriptive statistics (mean, standard deviation, and frequency distributions) were conducted to show the demographic characteristics. A correlation matrix was calculated using partial correlation analysis for social support, depressive symptoms and QOL with demographic variables controlled. The mediating effect of depressive symptoms for social support and QOL was examined via a nonparametric bootstrapping procedure using the SPSS macro PROCESS (model 4) (http://www.afhayes.com) suggested by Hayes [41], which was not based on large-sample theory and made no assumptions about the shape of the distributions of the variables [42]. The number of bootstrap resamples was chosen to be 5000, under the bias corrected 95% confidence interval (CI).

**Fig. 1:**
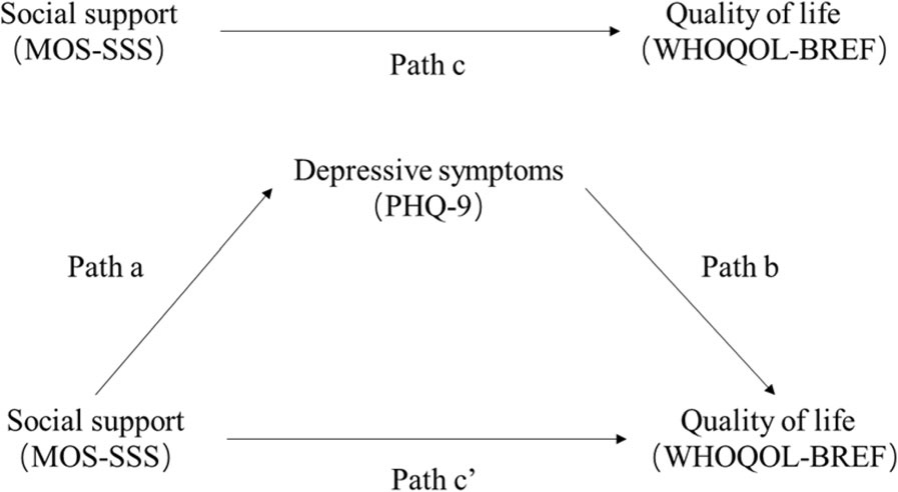
Diagram of paths in the mediation model of quality of life

The mediation analysis by PROCESS was based on regression-based path analysis. Path coefficients (a, b, c, and c’) in the mediation model were obtained (see Fig. 1). The a-path represents the relationship between predictor (social support) and mediator variables (depression). The b-path indicates the association between the mediator (depression) and outcome variables (QOL) while the predictor variable (social support) is controlled. The c’-path (also called “direct effect”) shows the relationship between the predictor and outcome variables excluding the mediator variable, while c-path (also named “total effect”) including the mediator variable [43]. The mediation effect (c-c’ = ab, ab was also known as “indirect effect”) [44] is indicated by a statistically significant difference between c and c’. The indirect effect would be significant with CIs not including zero [42].

Actually, it can be said that none of the existing mediating effects can be satisfied, or that no single effect size can measure the size of mediating effects [45]. According to the recommendation of Wen and Fan [46], multiple statistics should be reported at the same time. We used percent mediation (P_M_, the ratio of the indirect effect to the total effect) and R-squared mediation (*R*^*2*^_*med*_) [47] as measures of effect size. P_M_ could be interpreted as the percent of the total effect accounted for by the indirect effect [48, 49]. Although it is unstable in several parameter combinations, and has excess bias in small sample sizes [47, 50], it is meaningful for a basic mediation model where the indirect effect ab and the direct effect c’ have the same sign, accompanied by the total effect and indirect effect in standardized form [45]. *R*^*2*^_*med*_ means the variance of Y (QOL) can only be explained by X (social support) and M (depressive symptoms) together, but not by X or M independently. Fairchild, MacKinnon [47] proved that when the sample size is greater than 50, the value of *R*^*2*^_*med*_ is stable and the deviation is small. The disadvantage of *R*^*2*^_*med*_ is that there may be negative values in some cases [51, 52]. Then we repeated the earlier mediation analyses with different dimensions of social support to investigate whether mediation effects were showed in different dimensions of social support. All analyses were performed in SPSS version 22.0.

## Results

### Characteristics of elderly in rural China

Table 1 displays the demographic characteristics of the sample.

**Table 1:** Demographic characteristics of elderly (*N* = 420)

Variable	n (%)
Age (range:60–96, M = 74.98, SD = 8.29)	
60–64	54 (12.9)
65–69	78 (18.6)
70–74	66 (15.7)
75–79	77 (18.3)
80–84	92 (21.9)
≥ 85	53 (12.6)
Gender	
Male	168 (40.0)
Female	252 (60.0)
Years of schooling	
Not educated	258 (61.4)
1–5 years	135 (32.1)
6–8 years	22 (5.2)
9–11 years	3 (0.7)
≥ 12 years	2 (0.5)
Marital status	
Married	248 (59.0)
Widowed	157 (37.4)
Single/separated/divorced	15 (3.6)
Economic satisfactory	
Very adequate	4 (1.0)
Adequate	53 (12.6)
Ordinary	269 (64.0)
Inadequate	86 (20.5)
Very inadequate	8 (1.9)

### Correlations between social support, depression and quality of life

We examined correlations among variables for magnitude and plausibility with regard to our hypothesis (Table 2). Social support was correlated negatively with depressive symptoms and positively with QOL. QOL was negatively significantly associated with depressive symptoms.

**Table 2:** Correlations and descriptive statistics of study variables (*N* = 420)

	MOS-SSS	Emotional/ informational support	Tangible support	Positive social interaction	Affectionate support	PHQ-9	Mean	Standard deviation
MOS-SSS	–						53.6	9.1
Emotional/ informational support	0.786**	–					12.7	2.7
Tangible support	0.911**	0.565**	–				22.0	4.0
Positive social interaction	0.847**	0.498**	0.728**	–			10.6	2.1
Affectionate support	0.851**	0.631**	0.669**	0.733**	–		8.3	1.7
PHQ-9	− 0.152**	− 0.105*	− 0.132**	− 0.139**	− 0.156**	–	7.4	2.9
WHOQOL-BREF	0.449**	0.414**	0.330**	0.397**	0.449**	−0.321**	44.0	4.3

*Notes.* **Correlation is significant at *p* < 0.01 (two-sided). *Correlation is significant at *p* < 0.05 (two-sided). MOS-SSS: Medical Outcomes Study Social Support Survey. The total score of MOS-SSS was used to evaluate the social support, and four dimension scores of MOS-SSS were used to assess different types of social support

### Mediation of depressive symptoms

The main results generated by SPSS macro PROCESS were presented in Table 3. Increased depressive symptoms were significantly associated with worse social support (*β* = − 0.0569; 95% CI: [− 0.0912, − 0.0226]) and QOL (*β* = − 0.3741; 95% CI: [− 0.4926, − 0.2555]). Social support significantly influenced QOL (*β* = 0.2232; 95% CI: [0.1797, 0.2667]), and this association were still significant after taking depressive symptoms into the model (*β* = 0.2232; 95% CI: [0.1797, 0.2667]).

**Table 3:** Depressive symptoms mediate the relationship between social support and QOL, with age, gender, educational level, marital status, and economic satisfactory as covariates (*N* = 420)

	Path a	Path b	Path c	Path c’	Indirect effect	Standardized indirect effect	*P*_*M*_	*R*^*2*^_*med*_
Mediation analysis 1: MOS-SSS								
*β*	−0.0569	− 0.3741	0.2232	0.2019	0.0213	0.0427	0.0954	0.0479
LLCI	−0.0912	−0.4926	0.1797	0.1597	0.0071	0.0145	0.0308	0.0188
ULCI	−0.0226	−0.2555	0.2667	0.2441	0.0421	0.0816	0.1934	0.0879
Mediation analysis 2: Emotional/informational support								
*β*	−0.1313	−0.4086	0.6778	0.6241	0.0536	0.0317	0.0791	0.0351
LLCI	−0.2483	−0.5283	0.5265	0.4796	0.0059	0.0032	0.0094	0.0106
ULCI	−0.0142	−0.2888	0.8290	0.7687	0.1154	0.0663	0.1713	0.0684
Mediation analysis 3: Tangible support								
*β*	−0.1095	−0.4098	0.3690	0.3241	0.0449	0.0399	0.1216	0.0336
LLCI	−0.1871	−0.5341	0.2652	0.2243	0.0105	0.0095	0.0095	0.0124
ULCI	−0.0319	−0.2855	0.4728	0.4240	0.0919	0.0783	0.0783	0.0661
Mediation analysis 4: Positive social interaction								
*β*	−0.2191	−0.3921	0.8409	0.7549	0.0859	0.0406	0.1022	0.0388
LLCI	−0.3648	−0.5131	0.6515	0.5723	0.0245	0.0123	0.0292	0.0129
ULCI	−0.0735	−0.2711	1.0302	0.9376	0.1721	0.0799	0.2134	0.0768
Mediation analysis 5: Affection support								
β	−0.2933	−0.3705	1.1202	1.0115	0.1081	0.0437	0.0970	0.0482
LLCI	−0.4639	−0.489	0.9037	0.8013	0.0403	0.0163	0.0356	0.0195
ULCI	−0.1227	−0.2519	1.3367	1.2218	0.2080	0.0809	0.1898	0.0876

*Notes.* There are four mediation analyses in the table, different in social support scores. Mediation analysis 1 uses the total score of social support, while other mediation analyses use dimension scores of MOS-SSS respectively. *P*_*M*_ Percent mediation, *R*^*2*^_*med*_ R-squared mediation. *LL* low limit, *CI* confidence interval, *UL* upper limit

There was a significant indirect effect on QOL by social support through depressive symptoms (ab = 0.0213; 95% CI [0.0071, 0.0421]), and the mediator accounted for approximately 9.5% of the total effect. The *R*^*2*^_*med*_ value of 0.0479 was indicates that slightly less than 4.8% of the variance in QOL was attributable to the indirect effect of social support through depressive symptoms. Applying Cohen’s [53] benchmark values for R^2^Δ (i.e., .02, .13, and 26), the effect size was in the small range.

Stratified analyses by different components of social support showed significant mediation effects. For emotional/informational support (ab = 0.0536; 95% CI [0.0059, 0.1154]), the mediator accounted for 7.9% of the total effect and approximately 3.5% of the variance in QOL was attributable to the indirect effect. For tangible support (ab = 0.0449; 95% CI [0.0105, 0.0919]), the mediator accounted for 12.2% of the total effect and 3.4% of the variance in QOL was attributable to the indirect effect. For positive social interaction (ab = 0.0859; 95% CI [0.0245, 0.1721]), the mediator accounted for 8.6% of the total effect and 3.9% of the variance in QOL was attributable to the indirect effect. For affectionate support (ab = 0.1081; 95% CI [0.0403, 0.2080]), the mediator accounted for 9.7% of the total effect and 4.8% of the variance in QOL was attributable to the indirect effect.

These results revealed that depressive symptoms mediated the association between social support and QOL, which was consistent with the hypothesis. Different types of social support had different impacts on QOL.

## Discussion

The study aimed to verify the mediating role of depressive symptoms in the relationship between social support and quality of life. Our results manifested that depressive symptoms mediated the association between social support and QOL in Chinese rural elderly. It provided evidences to support that social support may influence QOL through psychological factors. In our analyses, each aspect of social support had impact QOL, and the relationships were mediated by depressive symptoms.

To our knowledge, previous studies mostly focused on depression as a mediator between social support and QOL among patients with HIV/AIDS [32, 54]. However, sociodemographic and medical differences between the samples of HIV/AIDS patients and our sample limit direct migration of these results. The present study is the first attempt to investigate the association between social support and QOL with an emphasis on the mediating role of depressive symptoms in the Chinese aging population.

To explain these findings, the cognitive appraisal theory is a potential approach [55]. In this theory, cognitive appraisal processes include primary appraisal, in which one evaluates the situation’s potential for harm and benefit, and secondary appraisal, in which one assesses the situation’s controllability and one’s available coping resources. According to the theory, it can be posited that a lack of social support leads to negative psychological states such as anxiety or depression, because of a primary appraisal of stressors, in the case of multiple illnesses with aging, and a secondary appraisal of coping resources. Conversely, the perception of good social support could balance the harmful primary appraisal, resulting lower level of depression. In turn, these psychological states may ultimately influence QOL either through a direct effect on physiological processes that influence susceptibility to disease or through behavioral patterns that increase risk for disease [8].

The unique contribution of the study lies in the finding that depressive symptoms partially mediate the effect of social support on QOL among rural elderly. That is, rural older people with poor social support are at higher risk of developing depression, which contribute to poor QOL [12, 56] and higher risk of suicide [57, 58]. In this study, the variance in QOL explained by depressive symptoms was relatively small. We think the reason is that the prevalence of depression in the assessed population was not high. Only 15.7% suffered moderate and even severe depression (the cut-off score of PHQ-9 was 10). Additionally, the partial mediation of depressive symptoms indicates that other variables probably mediated the association between social support and QOL, such as resilience [59]. Nevertheless, we couldn’t neglect the mediation effect of depressive symptoms. Insufficient social support decreases the QOL of elderly people, and depression caused by social isolation worsens the QOL and increases disease torture and economic burden [60].

An implication of the mediation effect is the importance of identification and treatment of depression, given the high risk of developing depression in aging population especially those with low social support. As the previous studies have indicated that older people in rural areas are short of mental health services [61], which could result in failure to obtain timely and adequate diagnosis and treatment of depression for older people in rural areas. Prevention of depression could have a profound impact on QOL and well-being of rural elderly. Indeed, the prevalence rates of depression were up to 12.7% by DSM IV and ICD 10, and 2.2–20.2% by other clinically based methods [62], making it the second most common chronic disorder for the elderly. However, depression among older adults is under-recognized in Chinese culture [63], resulting in poor QOL [17], an exacerbation of preexisting medical conditions [64], an increase the risk of suicide [57] and dementia [65].

To deal with the challenges of increasing burden of mental disorders, it would be better to incorporate social support in interventions targeting or tailoring older people who always suffered by depression in rural China. There maybe two reasons. Firstly, social isolation may prevent older people from seeking and receiving social support [66], which may aggravate depression and reduce quality of life. Secondly, social support interventions can promote the treatment adherence [67], contributing to enhancing the subjective feeling of elderly, and alleviating their anxiety, depression, and physical discomforts and pains, thus improving their quality of life.

Given that all four dimensions of social support were indicated to be associated to QOL, those who would provide social support for elderly could be trained to take corresponding methods regarding these aspects in the future. For example, to provide informational support, the village doctors and other stakeholders could improve skills about communications with older adults regarding their physical and mental health. To provide emotional and affection support, the village doctors and other stakeholders were encouraged to be more patient and careful when listening to the older people. Additionally, regular community-based activities and weekly visits to those living alone could provide tangible support and positive social interaction.

Several studies have suggested that depression influences perceived social support and subsequently QOL [68, 69], while this study supported that social support has an impact on depressive symptoms and subsequently QOL. Some investigators have termed this bidirectional association of two independent variables as “moderation”. In this study, other factors addition to social isolation causing depression cannot be ruled out. It is possible that depression caused by other factors has decreased social activities and affected perceived social support, and the reduction in social support has exacerbated depression, which ultimately led to a decline in QOL. Thus, it still needs more evidence to clarify these relationships.

This study also has some other limitations. First, the findings may not be generalized to other settings because the sample is only from two towns in a rural county of Hangzhou, and the sample size is small. However, because both depression and poor social support are common issues around the world, our findings may still provide insights to prevent and intervene depression and improve QOL in a wide range of cultures. Second, depression was assessed without clinical diagnose. Nevertheless, the PHQ-9 is a valid and effective measure of depressive symptom severity, which is highly correlated with diagnosis of major affective illness by the cutoff of 10 or more. It’s necessary to conduct a more complete diagnostic assessment of depression in the further research, for depression is a clinical concept. Third, the lack of measurement of related factors like the comorbidities of chronic disease, diet, exercise, Activity of daily living, financial resources and other related factors were limitations in understanding the associations of interest. Chronic diseases and Activity of daily living were closely related to QOL in elderly. Future researches are recommended to include these factors for more robust evidence. Fourth, there was a partial mediation of depressive symptoms on the relationship between social support and QOL. Future research needs to clarify and integrate further variables in a model of social support and QOL among rural elderly people. Finally, this was a cross-sectional study that collected data at a single time point. Future researches are recommended to examine these associations in longitudinal studies.

## Conclusions

Poor social support is significantly associated with the risk of depression and low QOL in rural elderly in China. Depressive symptoms significantly mediate the relationship between social support and QOL. These results suggest that social support is crucial to older adults’ health and QOL, and therefore it would be helpful for China’s rural elderly to incorporate social support in outpatient services afforded by village doctors or in community activities serviced by social workers.

## Data Availability

The datasets used and/or analyzed during the current study are available from the corresponding author on reasonable request.

## Abbreviations

QOL: Quality of life
MOS-SSS: Medical Outcomes Study Social Support Survey
